# Impact of pharmacogenomic *DPYD* variant guided dosing on toxicity in patients receiving fluoropyrimidines for gastrointestinal cancers in a high-volume tertiary centre

**DOI:** 10.1186/s12885-023-10857-8

**Published:** 2023-04-26

**Authors:** David K. Lau, Caroline Fong, Faten Arouri, Lillian Cortez, Hannah Katifi, Reyes Gonzalez-Exposito, Muhammad Bilal Razzaq, Su Li, Aislinn Macklin-Doherty, Monica Arenas Hernandez, Michael Hubank, Charlotte Fribbens, David Watkins, Sheela Rao, Ian Chau, David Cunningham, Naureen Starling

**Affiliations:** 1 Gastrointestinal and Lymphoma Unit, Royal Marsden NHS Foundation Trust, London and Sutton, UK; 2 Department of Pharmacy, Royal Marsden NHS Foundation Trust, London and Sutton, UK; 3 Purine Research Laboratory, Synnovis, St Thomas’ Hospital, London, UK; 4 Centre for Molecular Pathology, Royal Marsden Hospital and Institute of Cancer Research, Sutton, UK

**Keywords:** DPYD, Dihydropyrimidine dehydrogenase, Capecitabine, 5-fluorouracil, Pharmacogenomics

## Abstract

**Background:**

Dihydropyrimidine dehydrogenase (DPD) is a key enzyme in the metabolism of fluoropyrimidines. Variations in the encoding *DPYD* gene are associated with severe fluoropyrimidine toxicity and up-front dose reductions are recommended. We conducted a retrospective study to evaluate the impact of implementing *DPYD* variant testing for patients with gastrointestinal cancers in routine clinical practice in a high volume cancer centre in London, United Kingdom.

**Methods:**

Patients receiving fluoropyrimidine chemotherapy for gastrointestinal cancer prior to, and following the implementation of *DPYD* testing were identified retrospectively. After November 2018, patients were tested for *DPYD* variants c.1905+1G>A (DPYD*2A), c.2846A>T (*DPYD* rs67376798), c.1679T>G (DPYD*13), c.1236G>A (*DPYD* rs56038477), c.1601G>A (DPYD*4) prior to commencing fluoropyrimidines alone or in combination with other cytotoxics and/or radiotherapy. Patients with a *DPYD* heterozygous variant received an initial dose reduction of 25–50%. Toxicity by CTCAE v4.03 criteria was compared between *DPYD* heterozygous variant and wild type carriers.

**Results:**

Between 1^st^ December 2018 and 31^st^ July 2019, 370 patients who were fluoropyrimidine naïve underwent a *DPYD* genotyping test prior to receiving a capecitabine (*n* = 236, 63.8%) or 5FU (*n* = 134, 36.2%) containing chemotherapy regimen. 33 patients (8.8%) were heterozygous *DPYD* variant carriers and 337 (91.2%) were wild type. The most prevalent variants were c.1601G > A (*n* = 16) and c.1236G > A (*n* = 9). Mean relative dose intensity for the first dose was 54.2% (range 37.5–75%) for *DPYD* heterozygous carriers and 93.2% (42.9–100%) for *DPYD* wild type carriers. Overall grade 3 or worse toxicity was similar in *DPYD* variant carriers (4/33, 12.1%) as compared to wild-type carriers (89/337, 25.7%; *P* = 0.0924).

**Conclusions:**

Our study demonstrates successful routine *DPYD* mutation testing prior to the initiation of fluoropyrimidine chemotherapy with high uptake. In patients with *DPYD* heterozygous variants with pre-emptive dose reductions, high incidence of severe toxicity was not observed. Our data supports routine *DPYD* genotype testing prior to commencement of fluoropyrimidine chemotherapy.

## Introduction

Fluoropyrimidines are integral chemotherapy drugs in the treatment of gastrointestinal cancers [1, 2]. In European oncology practice, the fluoropyrimidines 5-fluorouracil (5FU) and the orally bioavailable capecitabine are amongst the most commonly drugs prescribed as systemic therapy for tumours arising from the lower gastrointestinal tract (colon, rectal, anal canal) and upper digestive tracts (oesophagogastric, hepatopancreatic biliary). Fluoropyrimidines are also commonly used in the management of head and neck [3] and breast cancers [4].

Severe fluoropyrimidine chemotherapy toxicity occurs in approximately 30% of recipients [5]. Toxicity is characterised by myelosuppression, gastrointestinal toxicity (including diarrhoea and mucositis), hand and foot syndrome and cardiac toxicity [6–8]. Mortality from severe fluoropyrimidine toxicity occurs in 0.1–0.5% of patients [7, 9].

Dihydropyrimidine dehydrogenase (DPD) is a critical protein in the enzymatic degradation of fluoropyrimidines. As the rate limiting step in fluoropyrimidine clearance, 5FU is converted to 5-dihydrofluorouracil by DPD in the liver and responsible for 80–85% of the catabolism of fluoropyrimidines to inactive metabolites [10]. Genetic variants in the corresponding encoding *DPYD* gene occur in approximately 8% of patients of Caucasian ethnicity. The variants DPYD*2A (c.1905+1G>A), c.2846A>T, DPYD*13 (c.1679T>G) and c.1236G>A are associated with attenuated enzymatic activity and more frequent fluoropyrimidine related adverse events [11, 12].

Following the pivotal study by Henricks et al. [13], which demonstrated pre-emptive fluoropyrimidine dose reductions based upon *DPYD* variants reduced severe toxicity, *DPYD* variant testing and pre-emptive fluoropyrimidine chemotherapy dose reduction was implemented by the Gastrointestinal Unit at the Royal Marsden Hospital in November 2018. In this study, we conducted an audit to assess the implementation of a *DPYD* variant guided dosing and its effect on severe toxicity.

## Methods

To identify patients receiving fluoropyrimidine chemotherapy, we interrogated the Royal Marsden Hospital pharmacy database for all prescriptions containing fluoropyrimidines (5FU and capecitabine) either in combination with other chemotherapy drugs and/or radiotherapy for gastrointestinal cancers after (1^st^ December 2018 to 30^th^ June 2019) *DPYD* testing implementation. Patients who had previously received systemic fluoropyrimidines were excluded from the study. To capture patients with homozygous *DPYD* variants, who may have not received fluoropyrimidines, we also searched for patients receiving raltitrexed, a non-fluoropyrimidine chemotherapeutic.

*DPYD* sequence variants were analysed in DNA extracted from EDTA whole blood. Common sequence variants *DPYD* c.1905+1G>A, c.2846A>T, c.1679T>G, c.1236G>A and c.1601G>A were genotyped by TaqMan assay (Applied Biosystems) using an AriaMx Real-Time PCR instrument (Agilent).

Treating physicians were provided with pathology reports which recommended initial dose reductions of 50% for heterozygous DPYD*2A or c.1679T>G variants; 25% for c.1236G>A and c.2846A>T; or 20% with a c.1601G>A variant. Patients with a *DPYD* heterozygous variant received an initial dose at the discretion of the treating physician. If initial doses of fluoropyrimidines were tolerated, doses of fluoropyrimdines were escalated for subsequent cycles. *DPYD* homozygous variant carriers did not receive fluoropyrimidines. *DPYD* wild-type carriers were dosed according to standard of care. Data on patient demographics, tumour, treatment and toxicity by CTCAE v4.03 criteria was collected retrospectively from electronic medical records, discharge summaries and chemotherapy charts.

The primary aim of the study was to compare the frequency of severe toxicity (grade ≥ 3) between *DPYD* wildtype and variant carriers with genotype based dosing. Other outcomes of interest were compliance with routine testing, turnaround time of *DPYD* testing, frequency of hospital admissions, treatment cessations and deaths due to fluoropyrimidine toxicity.

Comparisons of outcomes between groups were analysed using risk ratios and the Exact Fisher test. All *P* values were two-sided and a *P* value < 0.05 was considered statistically significant. Statistical analysis was performed on R-studio version 0.99.447.

### Patient and public involvement

Patients and the public were not involved in the design, or conduct, or reporting, or dissemination plans of this research.

## Results

**Fig. 1:**
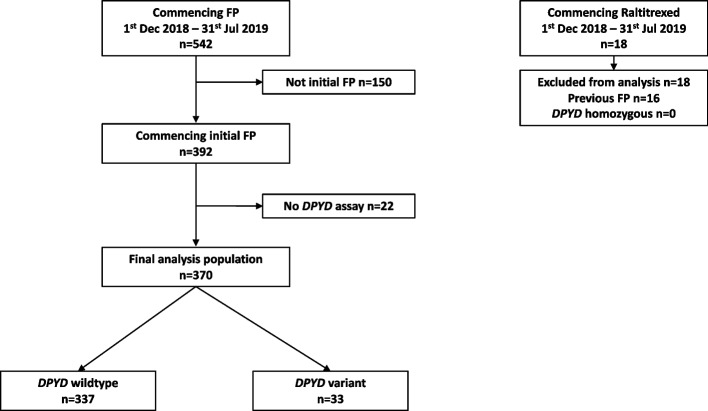
CONSORT diagram Abbreviations: FP – fluoropyrimidine, *DPYD* – dihydropyrimidine dehydrogenase

To compare the frequency of severe toxicities, we identified patients commencing fluoropyrimidine chemotherapy following implementation of routine *DPYD* testing in November 2018. Between 1^st^ December 2018 and 31^st^ July 2019, we identified 542 patients commencing fluoropyrimidine chemotherapy, of whom 150 patients were not receiving fluoropyrimidines for the first time. The remaining 392 patients were naïve to fluoropyrimidine chemotherapy. The *DPYD* guided cohort was comprised of the 370 patients (94.6%) who underwent, *DPYD* variant testing prior to receiving fluoropyrimidine containing chemotherapy. (Fig. 1).

Of the patients in the analysis (*n* = 370), the median age was 64 years (range 30–90). The majority were male (64.3%) and of white ethnicity (274, 74.1%). The predominant tumour type was colorectal cancer (*n* = 209, 56.5%), followed by oesophagogastric (*n* = 93, 25.1%) and hepatopancreatic biliary (*n* = 52, 14.1%). The majority of patients had an ECOG performance status of 0 (*n* = 100, 27%) or 1 (*n* = 254, 68.6%). The characteristics amongst the *DPYD* variant and wildtype cohorts were similar. (Table 1).

**Table 1:** Patient baseline characteristics

	*DPYD* variant N (%)	*DPYD* wildtype N (%)	Total N (%)
Total	33 (8.9)	337 (91.1)	370 (100)
Median age, years (range)	62 (30–88)	65 (28–90)	64 (28–90)
Sex			
Male	21 (63.6)	217 (64.4)	238 (64.3)
Female	12 (37.4)	120 (35.6)	132 (35.7)
Tumour			
Colorectal	23 (69.7)	186 (55.2)	209 (56.5)
Oesophagogastric	5 (15.2)	88 (26.1)	93 (25.1)
Hepatopancreatic biliary	4 (12.1)	48 (14.2)	52 (14.1)
Other ^a^	1 (3.0)	15 (4.5)	16 (4.3)
Ethnicity			
White	27 (81.8)	247 (73.1)	274 (74.1)
Asian	2 (6.1)	27 (8.0)	29 (7.8)
Black	0 (0)	15 (4.4)	15 (4.1)
Other	4 (12.1)	26 (7.7)	30 (8.1)
Undisclosed	0 (0)	12 (3.6)	12 (3.2)
ECOG Performance Status			
0	10 (30.3)	90 (26.6)	100 (27.0)
1	21 (63.6)	233 (68.9)	254 (68.6)
2	2 (6.1)	14 (4.1)	16 (4.3)

Abbreviations: *DPYD –* dihydropyrimidine dehydrogenase, ECOG – Eastern Co-operative Oncology Group. ^a ^includes ten patients with anal carcinoma, three patients with small bowel carcinoma, two patients with ampullary carcinoma and 1 patient with large cell neuroendocrine carcinoma

### *DPYD* testing

For *DPYD* testing, the median time from blood draw to result was 6 days (IQR 5–7, range 0–18). Twenty-two *DPYD* tests were missed, with the majority of the missed tests (17/22, 77.2%) occurring with the first 2 months of testing implementation.

Amongst the 370 patients who underwent *DPYD* genotyping, 36 variants were detected amongst 33 patients (8.9%). The most common variants were c.1601G>A (*n* = 16, 4.3%), followed by c.1236G>A (*n* = 11, 3.0%), c.1905+ 1G > A (*n* = 4, 1.1%), c.2846A>T (*n* = 4, 1.1%) and c.1679T >G (*n* = 1, 0.3%). Thirty patients had a single heterozygous variant and 3 patients had a compound heterozygous variant. All compound heterozygous variants were associated with the c.1601G>A variant which co-occurred in two patients with c.1236G > A, and one patient with c.1905+1G>A. Concurrently, we identified 18 patients who received raltitrexed following *DPYD* genotyping implementation, of which none were carriers of homozygous *DPYD* variants.

### Treatment

The majority of the patients were receiving fluoropyrimidine chemotherapy treatment with curative intent (*n* = 207, 55.9%) or as first line therapy (*n* = 359, 97%). Fluoropyrimidines were most commonly administered as a part of a chemotherapy doublet regimen (*n* = 219, 59.2%), single agent therapy (*n* = 92, 24.2%) or triplet therapy (*n* = 59, 15.9%). In 64 patients (17.3%), fluoropyrimidines were administered in combination with radiotherapy. Capecitabine was the most frequently prescribed fluoropyrimidine (*n* = 236, 63.8%), with the remainder of the patients receiving 5FU (*n* = 134, 36.2%). The proportions of characteristics were similar between the *DPYD* variant and wildtype cohorts. The relative dose intensities of the initial cycle of fluoropyrimidine was 54.2% (range 37.5–75%) for *DPYD* variant carriers, and 93.2% (42.9–100) for *DPYD* wildtype carriers. The two patients with compound heterozygous variants c.1236G>A/c.1601G>A commenced fluoropyrimidines at 50%. One patient with the compound heterozygous variant c.1905+1G>A/c.1601G>A was treated with an initial dose intensity of 41%. (Table 2).

**Table 2:** Characteristics of patients with *DPYD* variant results

	*DPYD* variant N (%)	*DPYD* wildtype N (%)	Total N (%)
Total	33 (8.9)	337 (91.1)	370 (100)
Treatment intent			
Curative	19 (57.6)	188 (5.6)	207 (55.9)
Palliative	14 (42.4)	149 (44.1)	153 (41.4)
Line of treatment			
First line	33 (100)	326 (96.4)	359 (97.0)
Second line	0 (0)	11 (3.3)	11 (3.0)
Median BSA m^2^ (25–75% IQR)	1.9 (1.8–2.1)	1.9 (1.7–2.0)	1.9 (1.7–2.0)
Mean relative initial dose intensity (range)	54.2% (37.5–75)	93.2% (42.9–100)	89.6% (38–100)
*c.1905*+*1G* > *A (n* = *3)*	50% (50–50)		
*c.2846A*> *T (n* = *4)*	50% (50–50)		
*c.1679T* > *G (n* = *1)*	50%		
*c.1236G*> *A (n* = *9)*	58.3% (50–75)		
*c.1601G*> *A (n* = *13) *^a^	53.1% (37.5–75%)		
Chemotherapy			
Single agent	9 (27.3)	83 (24.6)	92 (24.9)
Doublet	21 (63.6)	198 (58.6)	219 (59.2)
Triplet	3 (9.1)	56 (16.6)	59 (15.9)
Anti-VEGF	0 (0)	6 (1.8)	6 (1.6)
Anti-EGFR	1 (3)	19 (5.6)	20 (5.4)
Trastuzumab	1 (3)	5 (1.5)	6 (1.6)
Anti-PD1/PDL1	0 (0)	6 (1.8)	6 (1.6)
Chemoradiotherapy	6 (18.2)	58 (17.2)	64 (17.3)
Fluoropyrimidine			
5FU	8 (24.2)	126 (37.4)	134 (36.2)
Capecitabine	25 (75.8)	211 (62.6)	236 (63.8)

Abbreviations: *DPYD –* dihydropyrimidine dehydrogenase, ECOG – Eastern Co-operative Oncology Group, BSA – body surface area, 5FU – 5-Fluorouracil. ^a ^Not inclusive of compound heterozygotes. Compound heterozygotes: 2 patients with c.1236G>A/c.1601G>A commenced fluoropyrimidines at 50%. One patient with c.1905+ 1G>A/c.1601G>A commenced with an initial dose of 41%

### *DPYD* wildtype vs *DPYD* variants

To understand the impact of pharmacogenomic guided dosing on *DPYD* variant carriers, we compared the toxicities of wildtype and variant carriers. In total, 4 patients (12.1%) in the *DPYD* variant cohort experienced grade ≥ 3 toxicity with 2 patients experiencing gastrointestinal toxicity and 1 patient with severe haematological toxicity. By comparison, 89 patients in the wildtype group experienced grade ≥ 3 toxicity with haematological (46, 13.6%) and gastrointestinal (29, 8.6%) the most frequent. Eleven patients, all in the wildtype cohort experienced cardiac toxicity of any grade. There were no statistically significant differences in the frequency of grade ≥ 3 adverse events between *DPYD* variant and wildtype carriers. (Table 3) In keeping with the intended dosing strategy, 10 patients (30.3%) in the *DPYD* variant cohort had a dose escalation after the first cycle of fluoropyrimidines whereas only 7 patients (2.1%) of the wildtype cohort received a dose escalation (*P* < 0.00001). Dose reductions were more common within the wildtype cohort compared with the variant cohort (18.3% vs 3.0%, *P* = 0.0261). The frequency of early treatment discontinuation (within the first two cycles of therapy) was similar in the wildtype and variant cohorts (3.3% vs 6.1%, *P* = 0.3245). Fluoropyrimidine dose reductions within the first two cycles of chemotherapy was also similar between both groups (6.1% vs 10.7%, *P* = 0.2282).

**Table 3:** Grade ≥ 3 toxicities and fluoropyrimidine chemotherapy dose modifications by *DPYD* genotype

	*DPYD* variant N (%)	*DPYD* wildtype N (%)	Relative risk (95% CI)	Exact Fisher *P*-value
	*N* = 33	*N* = 337		
Grade ≥ 3 gastrointestinal	2 (6.3)	29 (8.6)	0.68 (0.17–2.73)	1
Grade ≥ 3 haematological	1 (3.1)	46 (13.6)	0.22 (0.03–1.53)	0.0998
Grade ≥ 3 dermatological	0 (0)	4 (1.2)	0 (0-N/A)	1
Any grade cardiac	0 (0)	11 (3.3)	0 (0-N/A)	0.609
Grade ≥ 3 other	1 (3.1)	15 (4.4)	0.64 (0.08–4.69)	1
Overall grade ≥ 3	4 (12.1)	89 (26.4)	0.46 (0.18–1.16)	0.0913
Deaths due to fluoropyrimidine toxicity	0 (0)	2 (0.6)	0.16 (0.02–1.13)	1
Dose modifications				
Dose reduction	1 (3.0)	62 (18.3)	0.16 (0.02–1.13)	0.0261
Dose escalations	10 (30.3)	7 (2.1)	12.80 (5.22–31.40)	< 0.00001
Cessation within first two cycles	2 (6.1)	11 (3.3)	1.70 (0.40–7.38)	0.3245
Dose reduction with first two cycles	2 (6.1)	36 (10.7)	0.55 (0.14–2.20)	0.2282
Patients requiring admission	4 (12.1)	46 (13.6)	0.87 (0.33–2.27)	1

Abbreviation(s): *DPYD –* dihydropyrimidine dehydrogenase

The clinical characteristics of the 4 carriers of *DPYD* variants experiencing severe fluoropyrimidine toxicity are detailed in Table 4. Three of these patients were receiving FOLFIRINOX (5FU, oxaliplatin, irinotecan) triplet chemotherapy which is frequency associated with severe toxicity [14]. In two patients, grade ≥ 3 toxicity occurred after 8 cycles of treatment which would be consistent with toxicity from non-fluoropyrimidine agents. Amongst *DPYD* wildtype carriers, grade ≥ 3 toxicity occurred in 10 patients (12.0%) receiving single agent chemotherapy and was significant higher in patients receiving doublet chemotherapy (53/198, 26.8%; *P* = 0.0074) and receiving triplet chemotherapy (26/56, 46.4%; *P* < 0.0001).

**Table 4:** Characteristics of patients with variant *DPYD* genotypes with severe toxicity

*DPYD* variant	Age (years)	Treatment	FP starting dose (%)	Toxicity
c.2846A > T het	60–69	FOLFIRINOX	50%	Grade 4 gastrointestinal toxicity after 1 cycle
c.1601G > A het	50–59	FOLFIRINOX	50%	Grade 3 other toxicity after 8 cycles
c.1601G > A het	60–69	CAPOX	75%	Grade 3 gastrointestinal toxicity after 2 cycles
c.1905 + 1G > A het	40–49	FOLFIRINOX	50%	Grade 4 haematological toxicity at 8 cycles

Abbreviations—*DPYD –* dihydropyrimidine dehydrogenase, FP – fluoropyrimdines, het – heterozygous, FOLFIRINOX – 5FU, leucovorin, irinotecan, oxaliplatin, CAPOX – capecitabine, oxaliplatin

## Discussion

Following the pivotal prospective study by Henricks et al. [13], we implemented routine *DPYD* pharmacogenomic guided dosing for patients commencing fluoropyrimidines for gastrointestinal cancers. Since initiating *DPYD* testing at the Royal Marsden Hospital, nationwide guidelines have been implemented with widely available *DPYD* genotype testing available through the NHS England Genomic Test Directory.

Our study demonstrates routine *DPYD* variant testing prior to the initiation of fluoropyrimidines can be successfully integrated into clinical practice. Compliance rates of *DPYD* testing was > 90% and the median laboratory turnaround time was 6 days. Patients were often tested for *DPYD* variants at the time of their initial medical oncology clinic appointment. Given the lead times for other investigations such as diagnostic imaging and chemotherapy day-unit scheduling, this turnaround time was not thought to cause a delay in the commencement of the chemotherapy. As the rollout of *DPYD* variant testing continues through the NHS England Genomic Test Directory, turnaround times are likely to improve. With the adoption of in-house testing, the turnaround time at our institution is now within 48 h.

The overall prevalence of the four *DPYD* variants recommended for testing (DPYD*2A, c.1236G>A, c.2846A>T, 1679G>A) amongst the population (20/330 5.4%) was slightly lower, but consistent with previous data in Caucasian populations. The c.1236A > T (HapB3) is the most prevalent clinically significant variant (2.4%), followed by c.1601G>A (DPYD*4) (2.0%), c.1905+1G>A (DPYD*2A) (0.8%), c.2846A>T (0.4%) and c.1679T>G (DPYD*13) (0.06%) [12]. The frequency of these variants are lower in other ethnic groups and further research is required to optimise a pharmacogenomic dosing approach in these populations [12].

In our clinical practice, we predominantly used 50% dose reductions of fluoropyrimidines with the first cycle of chemotherapy. Whilst this is recommended for heterozygous DPYD*2A and c.1679T >G variant carriers, we also extended this to carriers of other variants. We based this practice on prospective data suggesting 25% dose reductions are inadequate to mitigate toxicity with the c.1236G>A and c.2846A>T variants [13].

The incidence of grade ≥ 3 toxicity were not statistically different between patients with *DPYD* variants and wildtype. Taking into account the propensity for fluoropyrimidine toxicity in *DPYD* variant carriers, our result does suggest some level of effectiveness of upfront dose reductions. The incidence of severe fluoropyrimidine toxicity amongst carriers of *DPYD* variants was (4/33, 12.1%) and was similar to other real world case series of pharmacogenomic guided fluoropyrimidine dosing [15–17].

Despite pre-emptive fluoropyrimidine dose reductions, four patients with *DPYD* variants experienced severe toxicity due to the use of fluoropyrimidine with other cytotoxic drugs. Three of these patients were receiving FOLFIRINOX chemotherapy which is associated with high rates of severe toxicity [14]. Severe toxicity occurred in 25.7% *DPYD* wild type patients with doublet and 46.4% receiving triplet combination therapy. Whilst this may provide an argument for the limitations of *DPYD* variant testing, it does underline the need to develop pre-emptively strategies to reduce toxicity with fluoropyrimidine combination regimens. DPD phenotypic testing (uracil), which was not performed in this study may have identified additional patients at risk of fluoropyrimidine toxicity [18].

The most important limitation of our study is its retrospective design and sample size from a single institution. Only 33 (8.9%) carriers of *DPYD* variants were identified which is insufficient to provide a definitive estimate of the incidence of severe toxicities. Indeed, it is possible that the *DPYD* wildtype carriers have a different *DPYD* variant which was not detected with the available assays. Severe toxicity is also further confounded by the use of combination regimens which could be independent of *DPYD* variant status. Due to the low number of patients with *DPYD* variants, we could not perform meaningful analyses of populations at higher risk of toxicity including recipients of concurrent radiotherapy [19], female sex [20], age [21] and adjuvant/metastatic intent [22]. We did not record creatinine clearance which is a known risk factor particularly for capecitabine toxicity, however upfront reductions are routinely indicated in our practice in patients with lower creatinine clearance (30-50 mL/min). The reporting laboratory provided fluoropyrimidine dose reduction recommendations rather than the DPD activity score. [12] The use of the activity score provides user friendly dosing guidance particularly if multiple clinically significant *DPYD* variants are detected.

Following the implementation of pre-emptive *DPYD* variant testing, the routine testing of the c.1601G>A variant is no longer recommended. Whilst one case series has reported the c.1601G > A variant as a clinically significant predictor of fluoropyrimidine toxicity [23], this was not proven in a meta-analysis [11]. Furthermore, biochemical analysis suggests this variant does not result in loss of enzymatic activity [24].

In our study, we did not record the efficacy outcomes of the treatments. Due to the sample size and heterogeneity of the cohorts, it would have been insufficiently powered to demonstrate a conclusive result. A case–control study suggested a pre-emptive dose reduction strategy results in similar outcomes to patients with *DPYD* wildtype [25]. Furthermore, pharmacokinetic studies suggest AUC drug exposure with *DPYD* guided dosing is similar amongst variant and wildtype patients  [13]. Whilst further study is necessary, the strategy of dose escalation based upon tolerance, was successful in ten patients (30.3%), and mitigates these efficacy concerns.

In conclusion, our study demonstrates successful implementation of routine *DPYD* variant testing amongst fluoropyrimidine naïve patients with gastrointestinal cancers. Severe toxicity amongst *DPYD* variant carriers was not observed and had comparable rates of toxicity to *DPYD* variant carriers. Our data supports routine testing and pre-emptive dosing strategies which is now standardly done within the NHS.

## Data Availability

Data from this study can be made available to other researchers upon reasonable request and subject to data transfer agreements. Requests should be directed to NS.
